# Endoscopic endonasal translacerum approach for resection of petroclival chondrosarcoma

**DOI:** 10.3171/2020.4.FocusVid.19978

**Published:** 2020-04-01

**Authors:** Joao Paulo Almeida, Zachary Cappello, Hamid Borghei-Razavi, Pablo F. Recinos, Raj Sindwani, Varun R. Kshettry

**Affiliations:** 1 Department of Neurosurgery, Neurologic Institute, Cleveland Clinic, Cleveland;; 2 Department of Otolaryngology, Head & Neck Institute, Cleveland Clinic, Cleveland;; 3 Department of Neurosurgery, Cleveland Clinic Florida, Weston, Florida; and; 4 Charlotte Eye, Ear, Nose and Throat Associated, Charlotte, North Carolina

**Keywords:** petrosectomy, transpetrous, chondrosarcoma, transnasal, skull base, internal carotid artery, ICA skeletonization, surgical video

## Abstract

Petroclival chondrosarcomas are a formidable surgical challenge given the close relationship to critical neurovascular structures. The endoscopic endonasal approach can be utilized for many petroclival chondrosarcomas. However, tumors that extend to the inferior petrous apex require working behind the internal carotid artery (ICA). We present a case of a 33-year-old with a 1-year history of complete abducens palsy, with imaging showing an enhancing mass centered at the left petroclival fissure and inferior petrous apex behind the paraclival carotid artery and extending down into the nasopharynx abutting the cervical ICA. In this video, we describe the surgical steps of the endoscopic endonasal translacerum approach with ICA skeletonization and mobilization. We also highlight the relevant surgical anatomy with anatomical dissections to supplement the surgical video. The patient did well without complications. Postoperative MRI demonstrated complete resection and pathology revealed grade II chondrosarcoma. He underwent adjuvant proton beam radiotherapy.

The video can be found here: https://youtu.be/80QXALJW9ME.

## Transcript

This video demonstrates the endoscopic endonasal translacerum approach for resection for petroclival chondrosarcoma.

**0:29** Thirty-three-year-old man presented with 1 year of complete left abducens palsy, without any other neurological symptoms.

**0:38** MRI demonstrated an enhancing mass behind the left paraclival ICA. The mass is centered at the left petroclival fissure and behind the lacerum segment of the ICA. The tumor extended down into the nasopharynx and abutted the left cervical ICA and jugular foramen. Here is the sagittal T1 and CISS. Here is a coronal CISS sequence demonstrating the relationship with the relevant segments of the internal carotid artery and the jugular bulb.

**1:11** Here is an outline of the surgical steps. A right-side nasoseptal flap is harvested and tucked into the maxillary sinus. We harvested fascia lata and local fat from the right leg. The nasal approach included left middle turbinectomy and ethmoidectomy, bilateral sphenoidotomy, and posterior septectomy. A lateral nasal approach begins with left maxillary antrostomy and identification and ligation of the SPA [sphenopalatine artery]. The video demonstrates the subsequent steps. Neuromonitoring was used for left abducens EMG and EEG for cerebral perfusion.

**1:49** The SPA has been ligated and the sphenoid process of the palatine bone has been removed. Here we have identified the pterygoid base and the vidian nerve. We will sacrifice the vidian nerve and that will allow aggressive drilling of the pterygoid base. The vidian nerve serves as a good axial landmark for the foramen lacerum. As we continue to drill the pterygoid base, the foramen lacerum will come into view. Our goal is to continue to drill until we reach the pterygosphenoid fissure.

**2:34** We made a nasopharyngeal incision respecting the eustachian tube. And our next step will be to drill the floor of the sphenoid. In general, this step is necessary to open up the space into the infrapetrous region. And in this specific case, it is necessary to access the nasopharyngeal portion of the tumor.

**2:58** Next step will be drilling of the clival bone. Bone wax can be used for hemostasis from the marrow spaces of the clivus. As we drill laterally toward the foramen lacerum, we begin to encounter the inferomedial portion of the petrous apex. This anatomical illustration demonstrates the relationship between the foramen lacerum, the petrous apex, the sphenoid floor, and, importantly, the eustachian tube.

**3:34** The next surgical step is full skeletonization of the paraclival and lacerum segments of the internal carotid artery. It is typically easier to do this by exposing the sella, working laterally to expose the cavernous sinus and the clinoidal carotid artery, and then working inferiorly to expose the paraclival carotid. As we work inferiorly, the bone can be progressively thinned with the drill until it can be removed with a Kerrison rongeur. The floor of the sella is removed as well as the bone overlying the inferior aspect of the cavernous sinus, which can be open in cases of tumor invasion.

**4:40** After we have exposed the lacerum segment of the internal carotid artery, we can finalize aggressive drilling of the remaining bone at the petrosphenoid fissure. This exposes the fibrocartilage that connects the foramen lacerum to the eustachian tube. The anatomical dissection on the left illustrates the fibrocartilage that connects the foramen lacerum to the eustachian tube. The dissection on the right illustrates how disconnection of the fibrocartilage is necessary to unlock the lacerum and infrapetrous surgical corridors. After we Doppler the safe area, we can use a needle-tip cautery to disconnect the cartilage. This exposes some tumor but also bone of the inferior petrous apex, which we will remove.

**5:52** Tumor resection begins and we first address the inferior aspect of the tumor in the nasopharynx. Using an angle endoscope, we are able to inspect the cavity for any tumor remnants. We are also able to Doppler the cervical ICA. We then went after the petroclival portion of the tumor. As tumor is removed, further bone of the inferior petrous apex is aggressively drilled. We also work to remove the remaining lateral clival bone located behind the paraclival carotid. The sella floor and upper clivus are drilled for maximum tumor margins. Here is the portion of the tumor that was intradural but largely respected the arachnoid. Behind the paraclival carotid, there is a triangular wedge-shaped piece of bone, the petrosal process of the sphenoid bone. There are dura attachments laterally which need to be dissected out to allow removal. In this case, this piece of bone was positive for focal invasion of chondrosarcoma. With an angle endoscope and a covered shaft drill, we are using the shaft of the drill to gently retract the paraclival carotid and drill a wide margin of medial petrous bone. We were able to inspect the inferior portion of the cavernous sinus, which is not violated by tumor, to remove a wide margin of the surrounding bone.

**8:37** For reconstruction, since the arachnoid was largely intact, we used a single onlay fascia lata, countersunk by a small piece of fat, followed by a nasoseptal flap. This was covered with dura sealant and nasopore.

**9:00** These are pre- and postop MRI demonstrating complete resection.

**9:06** The patient did well, without complications, and pathology revealed a grade II chondrosarcoma. At 2 months postop, the patient reported partial improvement of abducens palsy. Adjuvant radiation versus observation were discussed and the patient opted for adjuvant proton beam.
